# *Rosa rugosa* R2R3-MYB transcription factors RrMYB12 and RrMYB111 regulate the accumulation of flavonols and anthocyanins

**DOI:** 10.3389/fpls.2024.1477278

**Published:** 2024-12-17

**Authors:** Yufeng Shi, Taoran Lu, Sanyan Lai, Song Li, Ling Zhang, Rong Liu, Lin Ouyang, Xinxin Zhao, Yuqin Jiang, Zhen Yan, Ju Zhang, Baohe Miao

**Affiliations:** ^1^ Institute of Urban Agriculture, Chinese Academy of Agricultural Sciences, Chengdu, China; ^2^ Chengdu National Agricultural Science and Technology Center, Chengdu, China; ^3^ State Key Laboratory of Tea Plant Biology and Utilization, Anhui Agricultural University, Hefei, China

**Keywords:** *Rosa rugosa*, R2R3 MYB, gene regulation, flavonols, anthocyanin

## Abstract

Roses (*Rosa rugosa*) are a famous flower with high ornamental and economic value. But the petals of roses are usually pink and purple, which restricted its application in garden settings. Flavonols and anthocyanins are crucial secondary metabolites related to flower pigmentation in plants. While MYB transcription factors involved in the biosynthesis pathway of anthocyanins have been identified in roses, the functional characterization of the MYB transcription factor regulating flavonol synthesis in *R. rugosa* remains unexplored. In this study, we isolated and characterized the R2R3-MYB transcription factors RrMYB12 and RrMYB111 involved in regulation of the flavonol biosynthetic pathway from *R. rugosa*. The bioinformatics analysis indicated that both the RrMYB12 and RrMYB111 belong to the R2R3-MYB subgroup 7 family. qRT-PCR analysis showed that *RrMYB12* and *RrMYB111* were expressed at low levels in roots and flowers. And transactivation activity assay indicated that RrMYB12 and RrMYB111 were transcriptional activators. The overexpression of *RrMYB12* and *RrMYB111* in tobacco resulted in an elevation of flavonol levels and a reduction in anthocyanin levels in flowers due to the upregulation of structural genes involved in flavonol synthesis, while the biosynthesis genes for the anthocyanin pathway were significantly downregulated. The transient reporter assay demonstrated that RrMYB12 exhibited strong activation of the promoters of *RrCHS* and *RrFLS* in *Nicotiana benthamiana* leaves following transient transformation. Furthermore, it was observed that RrMYBs displayed binding specificity to the promoter region of *CsFLS*.The functional characterization of the flavonol synthesis regulatory factors RrMYB12 and RrMYB111 offers a deeper understanding of the regulatory mechanism governing flavonol biosynthesis in roses, while also presenting an effective tool for genetic manipulation aimed at creating new varieties.

## Introduction

Flavonoids, a diverse group of plant secondary metabolites, are widely distributed in natural plants. The types and concentrations of flavonoids vary among different plant species, with Flavonols and anthocyanins playing multifaceted roles in plant growth and defense (Liang et al., 2023; Liu et al., 2024; Shi et al., 2021). In addition to preventing biological and abiotic damage, they also impart rich colors to flowers and other organs (Ji et al., 2023; Zhou et al., 2023). The color range of flavonols primarily encompasses white or yellow, whereas anthocyanins span from red and purple to blue, among others. These pigments are synthesized within the flavonoid metabolic pathway, a branch of the phenylpropanoid metabolic pathway (Holme et al., 2021).

The biosynthetic pathways of flavonoids have been extensively studied in *Arabidopsis thaliana*, *Nicotiana tabacum*, *Camellia sinensis* and other plant species (Raziq et al., 2024; Saito et al., 2013; Wang et al., 2018). Almost all structural genes involved in the flavonoid biosynthesis pathways that have been isolated and identified to date are conserved in higher plants. The upstream pathway of flavonoid biosynthesis includes chalcone synthetase (CHS), chalcone isomerase (CHI), and flavonoid 3-hydroxylase (F3H). CHS plays a crucial role as the first step in the flavonoid biosynthesis pathway, producing naringin chalcone (Yin et al., 2019). Another important node is the diversion of dihydrokaempferol, which can be directed towards either flavonol biosynthesis or anthocyanin biosynthesis. Flavonol biosynthesis is a branch of the flavonoid biosynthesis pathway. Dihydroflavonols are synthesized into flavonols by flavonol synthase (FLS), and then various more stable flavonol glycosides are generated by flavonol glycosyltransferase (UGT). Anthocyanin biosynthesis is performed by dihydroflavonol 4-reductase (DFR), anthocyanin synthetase (ANS) and flavonoid 3-glucosyltransferase (UFGT).

Regulatory proteins are essential for the activation or inhibition of flavonoid biosynthesis, and extensive research has consistently demonstrated that MYB, bHLH and WD40 transcription factors play a predominant role in mediating the transcriptional regulation of the flavonoid biosynthesis pathway. Studies have found that each specific branch of the flavonoid pathway is typically regulated by different MYB transcription factors. In *A. thaliana*, R2R3-MYBs are divided into 25 subfamilies based on differences in their C-terminal regions (Stracke et al., 2001). Studies have found that MYBs in subgroup 4 (SG4), subgroup 5 (SG5), subgroup 6 (SG6) and subgroup 7 (SG7) are involved in regulating the synthesis of flavonoids. Many MYB transcription factors, which regulate flavonoid biosynthesis pathways, have been identified from different plant species. The R2R3-MYB SG5 members, such as FaMYB5 (*Fragaria* × *ananassa*), EjMYB5 (*Eriobotrya japonica*), and JrMYB1(*Juglans regia*), exert positive regulatory effects on proanthocyanidin (PA) biosynthesis (Jiang et al., 2023; Zhang et al., 2024; Zhao et al., 2023). EpMYB1 (*Echinacea purpurea*) and RcMYB1 (*Rosa chinensis*) were in SG6, which is a group known to promote the accumulation of plant anthocyanins (He et al., 2023; Jin et al., 2024). And AtMYB12/AtMYB11/AtMYB11 (*Arabidopsis thaliana*), VvMYBF1 (*Vitis vinifera*) and PqMYBF1 (*Paeonia qiui*) were classified in SG7, which is a branch responsible for regulating the biosynthesis of flavonols (Stracke et al., 2007; Wang et al., 2020; Zhang et al., 2023).

In regulation of the anthocyanin and PA biosynthetic pathways, MYB transcription factors generally interact with basic helix-loop-helix (bHLH) transcription factors and WD-repeat (WDR) proteins to form a MYB-bHLH-WD40 (MBW) complex. For instance, in *R. chinensis*, two MBW complexes (RcMYB1-RcBHLH42-RcTTG1; RcMYB1-RcEGL1-RcTTG1) were identified that can enhance the promoter activity of RcMYB1 and late anthocyanin biosynthesis genes, thereby promoting anthocyanin accumulation (He et al., 2023). However, in regulation of the flavonol biosynthetic pathway, R2R3-MYB regulators identified to date are found to function independent of bHLH cofactor. These flavonol-specific MYB regulators can activate the promoters of target genes involved in flavonol biosynthesis, including *CHS*, *CHI*, *F3H* and *FLS*, and thus promote the synthesis of flavonols. The ectopic expression of *PqMYBF1* (*Paeonia qiui*), *EsMYBF1* (*Epimedium sagittatum*) and *GhMYB1a* (*Gerbera hybrida*) in transgenic tobacco resulted in a discernible elevation in flavonol levels, accompanied by a significant reduction in anthocyanin accumulation, leading to the development of flowers with a pale pink hue (Huang et al., 2016; Zhang et al., 2023; Zhong et al., 2020). Furthermore, the overexpression of *GhMYB1a* in gerbera led to a decrease in anthocyanin accumulation and an increase in flavonol accumulation by upregulating the structural genes involved in flavonol biosynthesis (Zhong et al., 2020). Dual-luciferase reporter assays showed that PqMYBF1 is capable of activating the promoters of *PqCHS*, *PqF3H*, and *PqFLS* (Zhang et al., 2023). GhMYB1 significantly activated the promoters of both *NtCHS* and *NtFLS*, but it did not activate the promoter of *GhDFR* compared with the corresponding controls (Zhong et al., 2020). Transient reporter assay showed that EsMYBF1 strongly activated the promoters of *EsF3H* and *EsFLS*, but not the promoters of *EsDFR*s and *EsANS* in transiently transformed *Nicotiana benthamiana* leaves (Huang et al., 2016). Both the yeast two-hybrid assay and the transient reporter assay validated that EsMYBF1 functions independently of EsTT8 or AtTT8, which are bHLH regulators of the flavonoid pathway, as cofactors.

Rose (*Rosa rugosa*) is a kind of important plant with ornamental and economic value, characterized by drought resistance, barrenness resistance, cold resistance, and wide adaptability to harsh natural environments (Chen et al., 2021; Xie et al., 2022; Zang et al., 2021). In the realm of ornamental plants, the commercial value of roses is significantly influenced by their vibrant array of colors. However, the monotonous flower colors of rose varieties have seriously restricted their application in gardens. The molecular mechanisms and transcription factors that control flower color in *R. rugosa* are not fully studied (Wang et al., 2022). To date, only RrMYB5 and RrMYB10 have been validated as regulators of anthocyanin and PA biosynthesis in *R. rugosa* (Shen et al., 2019). In this study, we report the in-depth characterization of R2R3-MYB transcription factors RrMYB12 and RrMYB111. Bioinformatics analysis shows that RrMYB12 and RrMYB111 belong to the SG7 R2R3-MYB. qRT-PCR results showed that *RrMYB12* and *RrMYB111* exhibited differences in tissue expression. In *RrMYB12*-OE and *RrMYB111*-OE transgenic tobacco, flavonol content increased, anthocyanin accumulation decreased, *NtCHS*, *NtCHI*, *NtF3H* and *NtFLS* genes were up-regulated, *NtDFR* and *NtANS* genes were down-regulated. In addition, RrMYB12 and RrMYB111 activated promoters of *RrCHS* and *RrFLS* genes were confirmed by protein-DNA interaction tests. These results indicate that RrMYB12 and RrMYB111 are flavonol specific R2R3-MYB regulatory factors involved in regulating flavonol biosynthesis in *R. rugosa*.

## Materials and methods

### Plant materials

Cultivars of the rose (*Rosa rugosa*) plant “Zizhi” were planted in nursery garden of Institute of Urban Agriculture, Chinese Academy of Agricultural Sciences (Chengdu, Sichuan, China), where the temperature was 26/20°C (day/night) and the a photoperiod in which there was 16/8 h (light/darkness). Tobacco G28 (*Nicotiana tabacum* cv. G28) and transgenic tobacco and *Nicotiana benthamiana* were grown in a growth chamber at a constant temperature of 24 ± 3°C and 12/12 h light/dark cycle.

### Gene clone of *RrMYB12* and *RrMYB111*

Total RNA was isolated from rose plants using the Fastpure Universal Plant Total RNA Isolation Kit (Vazyme, China). The experiment was performed according to the manufacturer’s instructions, followed by reverse transcription into cDNA using a PrimeScript RT Reagent Kit with gDNA Eraser (Takara, Japan). Gene was cloned by PrimeSTAR Max DNA Polymerase (Takara, Japan). Amplification of full length *RrMYB12* and *RrMYB111* cDNA with the open reading frame (ORF) primers listed in **Supplementary Table 1** was performed by PCR, after which the sequence was generated. The generated sequence was checked by BLASTX and BLASTp against GenBank, and the sequences of the homologs were retrieved to perform an alignment analysis.

### Bioinformatic analysis of RrMYB12 and RrMYB111

Theoretical molecular weights (MW) and isoelectric points (pI) were calculated using the Compute pI/Mw tool (http://us.expasy.org/tools/pi_tool.html). The amino acid sequence alignment analysis of RrMYB12 and RrMYB111 were conducted by using the DNAMAN software. A phylogenetic analysis was conducted using the amino acid sequences of R2R3-MYB members through MEGA7 software. Evolutionary distances were estimated using the *p*-distance method in the software, and the tree nodes were evaluated with a bootstrap value of 1000. In addition, the exon/intron genomic structure of *RrMYB12* and *RrMYB111* gene was analyzed by TBtools.

### Transcriptional activity analysis

The target vector *RrMYB12*-pGBKT7 and *RrMYB111*-pGBKT7 was constructed by ClonExpress II One Step Cloning Kit (Vazyme, China), and the primer sequences are shown in **Supplementary Table 1**. Empty vector of pGBKT7 was used as negative control. The construct (*RrMYB12*-pGBKT7, *RrMYB111*-pGBKT7) and empty BD vector (pGBKT7) were transformed into the yeast Y2H (Weidi, China) by Y2HGold Chemically Competent Cell product manual, respectively. Monoclonal yeast plaques of construct and empty plasmids were picked and dissolved in 50 µL ddH_2_O and adjusted to be OD600 = 0.2, respectively. And dilute these yeast cells by 10 times, 100 times, 1000 times, and 10000 times respectively. The dilutions were incubated on SD/-Trp, SD/-Trp-His, SD/-Trp-His + 20 µg/mL X-α-Gal medium at 30 °C for about 72 h to observe the formation of plaque and coloration.

### Quantitative real-time PCR

Rose plants various tissues samples and tobacco flowers samples were frozen at -80 °C and ground in liquid nitrogen. Total RNA was extracted from the samples using an RNA extraction kit (Vazyme, China). Reverse transcription was performed using M5 Super plus qPCR RT kit with gDNA remover (Mei5bio, China) using 1000 ng cDNA in a 20 μl reaction volume. After reverse transcription, the cDNA was diluted to 25% (v/v) with deionized water before being used as the template. Gene expression was detected in 1 μl cDNA using an M5 HiPer Real-time PCR mix (Mei5bio, China) and the quantstudio 1 system (Thermo Fisher Scientifc, USA). *RrGAPDH* and *Ntactin* were used as reference genes (Jiang et al., 2020; Shen et al., 2019). All of the above experiments consisted of at least seven biological and three technical replicates. The related primers are shown in **Supplementary Table 4**.

### Plasmid construction and heterologous expression of *RrMYB12* and *RrMYB111* in *N. tabacum*

The Gateway^®^ cloning system (Invitrogen, USA) was used to construct the vectors provided by state key laboratory of tea plant biology and utilization (Jiang et al., 2020). To generate vectors for the overexpression of *RrMYB12* and *RrMYB111*, the PCR products were purified and cloned into the entry vector pDONR207 by using a Gateway BP clonase enzyme mixture (Invitrogen, USA) according to the manufacturer’s instruction with the primers listed in **Supplementary Table 1**. And transfer the positive plasmid into into the expression vector pCB2004 by using the Gateway LR clonase system (Invitrogen, USA) according to the manufacturer’s instruction. The recombinant pCB2004-*RrMYB12* and pCB2004-*RrMYB111* plasmids were transferred into *Agrobacterium tumefaciens* GV3101 (Weidi, China) by GV3101 Chemically Competent Cell product manual. Wild-type plants of tobacco G28 were used for the *A. tumefaciens*-mediated transformation. The leaf-disk approach was used for tobacco transformation by adding 10 mg/L of glufosinate ammonium for selection.

### Metabolic analysis of phenolic compounds in tobacco flowers

The anthocyanins and flavonol glycosides were extracted from tobacco flowers and quantified as proposed by Jiang et al (Iang et al., 2018; Jiang et al., 2020). The quantitative detection of the compounds was performed using the MRM mode of the Agilent 6460 QQQ-MS/MS LC system (Agilent Technologies, USA).

### Promoter clone and analysis

Genome DNA was isolated from rose plants using the FlaPure Plant DNA Extraction Kit (Genesand, China). Promoter sequence was cloned by PrimeSTAR Max DNA Polymerase (Takara, Japan), and the sequence primers were listed in **Supplementary Table 2**. Cis-acting elements involved in MYB binding in the gene promoters were analysed using the PlantCARE database (http://bioinformatics.psb.ugent.be/webtools/plantcare/html/).

### Electrophoretic mobility shift assay

The complete coding sequence of *RrMYB12* and *RrMYB111* was cloned into pMAL-c2X, respectively for prokaryotic expression of the fusion protein. The newly constructed vector was transformed into *Escherichia coli* strain BL21. To induce protein expression, 0.4 mM isopropyl β-d-1-thiogalactopyranoside was added to *E. coli* cultures in LB medium, followed by incubation at 28 °C for 24 h. The *E. coli* cells were centrifuged at 4500 rpm/min at 4 °C for 12 min, and the LB medium was removed. The *E. coli* cells were resuspended in equilibration buffer (EB supplemented with 20 mM Tris-HCl, 200 mM NaCl, 1 mM EDTA, pH7.4, and 1 mM DTT dithiothreitol for ultrasonic cell disruption. The *RrMYB12*-pMAL and *RrMYB111*-pMAL protein was purifed with Maltose binding protein (MBP) (New England Biolabs, USA). The *RrMYB12*-pMAL and *RrMYB111*-pMAL and No-load protein was bound to the amylose resin using the equilibration buffer, respectively. The *RrMYB12*-pMAL and *RrMYB111*-pMAL protein was then eluted using a concentration of 100 mM maltose.

The probes were labeled with biotin and annealed to form double-stranded DNA, Sequences of probes used for EMSA were listed in **Supplementary Table 3**. For EMSA experiments, The concentration of the *RrMYB12*-pMAL and *RrMYB111*-pMAL protein was maintained at 1 mg/ml, and 2 µl was added to each experimental group. The biotin-labeled probe concentration was set at 10 μM. Positively chgd. nylon transfer membrane (GE Healthcare, USA) was used for the transmembrane step. EMSAs were performed with the Chemiluminescent EMSA Kit (Beyotime Biotechnology, China) according to the manufacturer’s instructions.

### Dual-luciferase reporter assay

The promoter fragments of *RrFLS* were inserted into the pGreenII0800-LUC vector as reporter constructs. The coding sequence of *RrMYB12* and *RrMYB111* was inserted in the pGreenII62-SK vector as the efector, respectively. All primers used for reporter and effector constructions were listed in **Supplementary Table 1**. All the reporter and effector constructs recombinant plasmids were transformed into *Agrobacterium tumefaciens* GV3101(pSoup-p19) (Weidi, China). *A. tumefaciens* was cultured on LB agar supplemented with selection antibiotics and incubated at 28 °C for 2 days. *A. tumefaciens* was expanded and centrifuged and resuspended in infiltration buffer (10 mM MES, 10 mM MgCl2, 100 µM acetosyringone) to an OD600 of 0.8. And *A. tumefaciens* solution was mised proportionally and incubated at room temperature with gentle shaking for 2-3 h before infiltration. Approximately 300 µL of this *A. tumefaciens* mixture was infiltrated into *Nicotiana benthamiana* young leaves of each plant. At least four plants were used for each treatment. After 3 days of incubation, D-Luciferin firefly, sodium salt monohydrate (Yeasen, China) was sprayed and fluorescence imaging was observed with NightSHADE evo *in vivo* plant imaging system (Berthold, Germany). And luminescence detected by using the Dual Luciferase Reporter Gene Assay Kit (Yeasen, China) and Spark^®^ multifunctional microplate detector System (Tecan, Swiss).

### Statistical analysis

SPSS software was used for statistical analysis. The datas of anthocyanin and flavonol glycosides and gene expression were detected at least three times independently, and all data are represented as the mean ± SD. Significant difference was determined using Student’s *t*-test significant difference test and indicated by the asterisk (*P < 0.05, **P < 0.01).

## Results

### Isolation and characterization of RrMYB12 and RrMYB111

**Figure 1:**
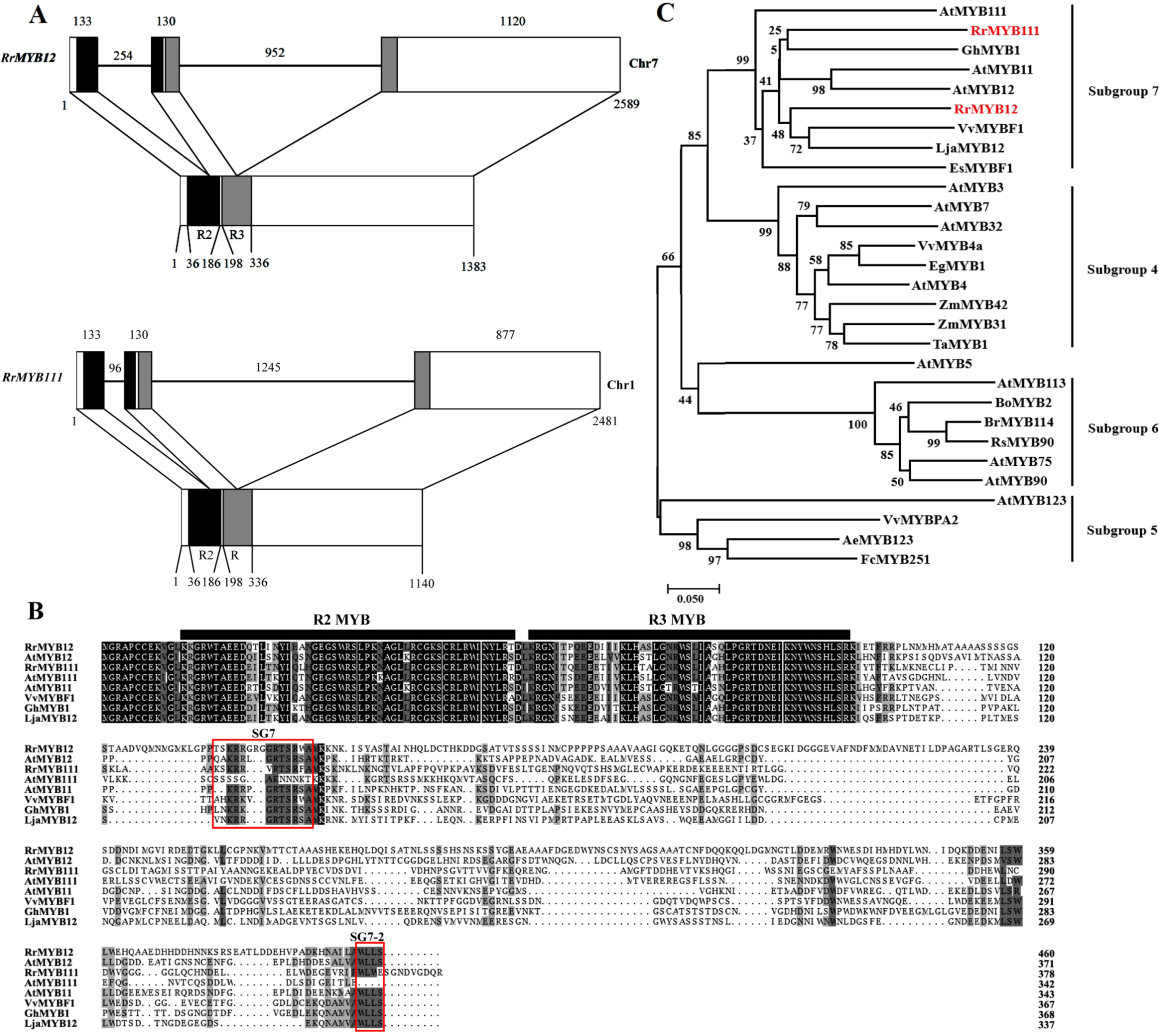
Sequence structure analysis and bioinformatic analysis of RrMYB12 and RrMYB111. **(A)** Genome sequence structure of *RrMYB12* and *RrMYB111.* The exons are shown as blocks and the introns as lines. The diagram top represents the genomic sequence, and the numbers located above show the length of exons and introns. The bottom of diagram means the cDNA sequence, and the numbers located below show the distance from the start codon of *RrMYB12* and *RrMYB111* gene. The R2 and R3 MYB domains are shown as black and gray blocks, respectively. **(B)** Alignment of the amino acid sequences of the RrMYB12 and RrMYB111. Identical amino acid residues are shaded in black, and similar in gray. The R2 and R3 MYB domainsare were marked with black bold horizontal line. Two conserved motifs, SG7 and SG7-2 were boxed in red box. **(C)** Phylogenetic analysis of RrMYB12, RrMYB111 and selected R2R3-MYB transcription factors from other plant species. The scale bar represents 0.05 substitution per site, RrMYB12 and RrMYB111 are marked in red. Sequence data from this article can be found in the NCBI database under the following accession numbers: RrMYB12 (XM_06213243), RrMYB111 (XM_062138075), AtMYB111 (NM_124310.3), AtMYB11 (NP_191820.1), AtMYB12 (NP_182268.1), VvMYBF1 (ACV81697), EsMYBF1 (ANZ79233.1), GhMYB1 (CACN01040333), LjaMYB12 (QER90717.1), AtMYB4 (NP_195574), AtMYB7 (NP_179263), AtMYB3 (NP_564176), AtMYB32 (NP_195225), VvMYB4a (NP_001268129.1), EgMYB1 (CAE09058), ZmMYB31 (AJ42202), ZmMYB42 (CAJ42204), TaMYB1 (AAT37167), AeMYB123 (XP_057475406), FcMYB251 (BAG75107), VvMYBPA2 (NP_001267953), AtMYB123 (NP_001331958), AtMYB5 (NP_187963), AtMYB90 (NP_176813), AtMYB75 (NP_176057), AtMYB113 (NP_176811), BoMYB2 (ADP76651), BrMYB114 (WEY29606.1), RsMYB90 (XP_018491141).

Two *RrMYB* gene sequences were retrieved from *Rosa rugosa* transcriptome database and genome database based on *A. thaliana* and *N. tabacum* SG7 MYB gene sequences. The full-length cDNA sequences of the two RrMYBs were obtained from the “Zizhi” cultivar, which is a special variety that blooms all year round, using high fidelity PCR. The two gene sequences had been uploaded to the National Center Biotechnology Information (NCBI, http://www.ncbi.nlm.nih.gov) database by someone else. The first sequence is named RrMYB12. The cDNA ORF length of the *RrMYB12* (accession number: XM_062132433) was 1383 bp, encoding a protein of 460 amino acids in length with a predicted pI value of 4.98. The second sequence is named RrMYB111. The cDNA ORF length of the *RrMYB111* (accession number: XM_062138075) was 1140 bp, encoding a protein of 379 amino acids in length with a predicted pI value of 5.99. Alignment between the genomic and cDNA sequences showed that the gene structures of the *RrMYB12* and *RrMYB111* in the genome database were similar, with each structure containing three exons and two introns (**Figure 1A**). The differences between the two *RrMYB*s were that their introns and the third exon lengths were different. These two introns were located into the R2 and R3 MYB domain, respectively. Moreover, the intron insertion places are highly conserved (**Supplementary Figure 1**).

The amino acid sequence analysis revealed that both RrMYB12 and RrMYB111 contained the R2 and R3 MYB DNA-binding domains in their N-terminus (**Figure 1B**). The similarity in amino acid sequences between MYB proteins is generally limited to the R2 and R3 repeats. High identity was observed between RrMYB12 and RrMYB111 with other MYB regulators within the R2 and R3 MYB domains, such as 84.31% and 87.25% identity with AtMYB12, and 83.33% and 88.24% identity with AtMYB111, respectively. However, within the overall protein sequence, RrMYB12 and RrMYB111 showed only 31.92% and 27.06% identity to AtMYB12, and 25.58% and 31.08% identity to AtMYB111, respectively. The SG7 motif ([K/R][R/x][R/K]xGRT[S/x][R/G]xx[M/x]K) characteristic of flavonol regulators was identified in the C-terminus of both RrMYB12 and RrMYB111 (**Figure 1B**). Besides, RrMYB12 also contains a similar counterpart (WLEE) of the SG7-2 motif ([W/x][L/x]LS) previously identified in the C-terminus, but RrMYB111 did not. Additionally, it is worth noting that the R2R3 repeat region of RrMYB12 and RrMYB111 does not contain the motif ([D/E]Lx2[R/K]x3Lx6Lx3R) for interaction with bHLH partners.

Phylogenetic analysis showed that RrMYB12 and RrMYB111 both belong to SG7 R2R3-MYB and clustered with other flavonol MYB regulators into a flavonol clade (**Figure 1C**). This suggests that RrMYB12 and RrMYB111 probably function as regulators of flavonol biosynthesis. Additionally, RrMYB111 was closely related to the AtMYB111, while RrMYB12 was closely related to the AtMYB12.

### Expression pattern of *RrMYB12* and *RrMYB111* genes in rose plants

**Figure 2:**
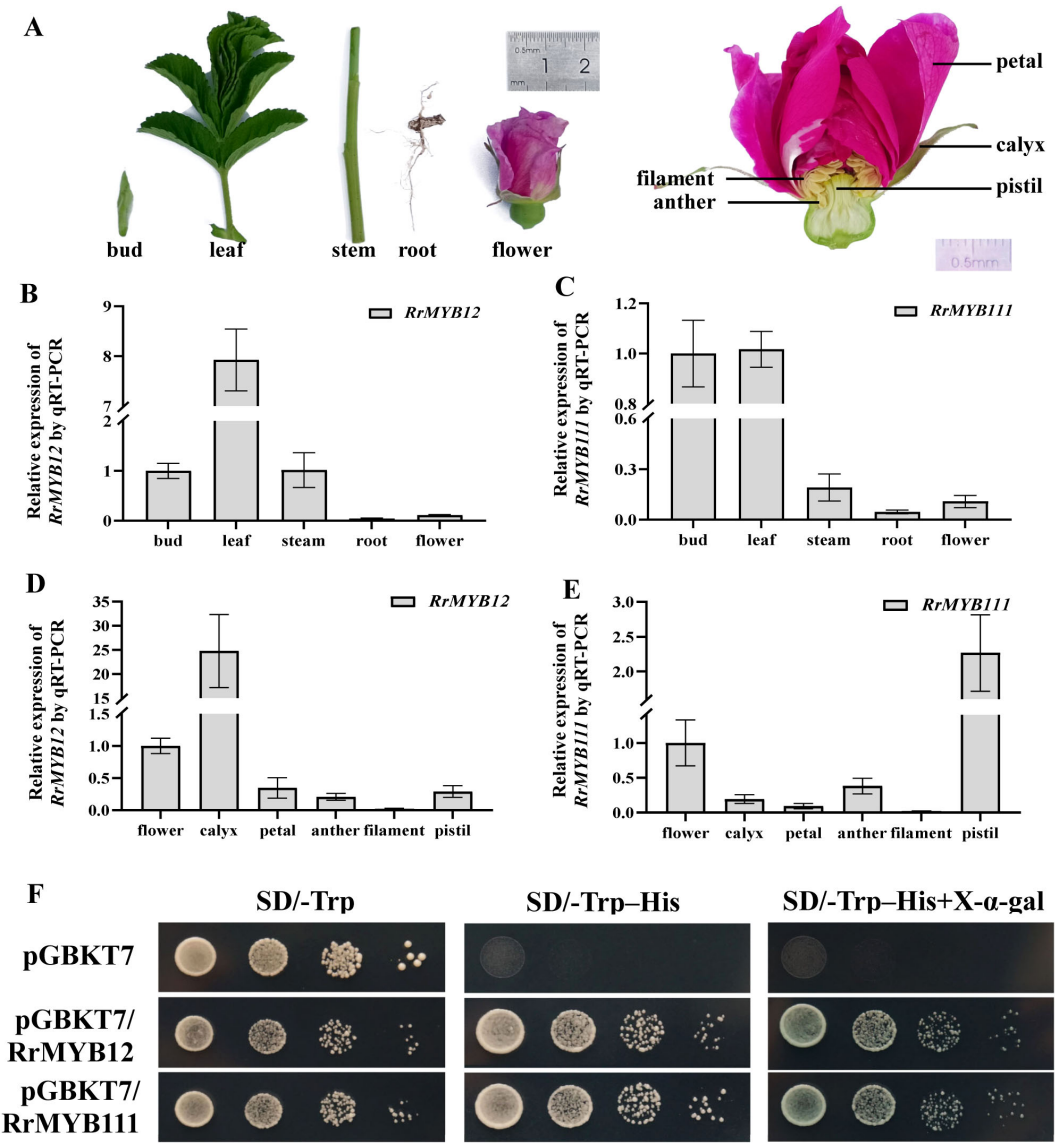
Expression levels and transcriptional activity of RrMYB12 and RrMYB111. **(A)** Tissues and organs phenotypes of rose plant materials used in this experiment. **(B, C)** *RrMYB12* and *RrMYB111* gene expression in different tissues and organs of rose plants detected by qRT-PCR. **(D, E)** *RrMYB12* and *RrMYB111* gene expression in different parts of the rose flower detected by qRT-PCR. **(F)** Transcriptional activator activity of RrMYB12 and RrMYB111 in yeast. pGBKT7 was used as the negative control.

The expression patterns of *RrMYB12* and *RrMYB111* in various tissues and organs of rose plants were analyzed by quantitative real-time PCR (qRT-PCR) (**Figure 2A**). The results showed that the expression profiles of both *RrMYB12* and *RrMYB111* were similarly characterized by low expression in root and flower, but *RrMYB12* was high expression in leaf and *RrMYB111* was high expression in young shoot (both bud and leaf)(**Figures 2B, C**). Additionally, considering the pivotal role of the rose flower in roses, thus we conducted an analysis on the expression patterns of *RrMYB12* and *RrMYB111* across different parts of rose flower (**Figure 2A**). The expression of *RrMYB12* and *RrMYB111* exhibited partial similarity and the expression of these two genes were relatively low in the petals and filaments. And the differences were that *RrMYB12* was highly expressed in the calyx, while *RrMYB111* was highly expressed in the pistil (**Figures 2D, E**).

### RrMYB12 and RrMYB111 function as transcriptional activator

We examined the transcriptional activation of RrMYB12 and RrMYB111 by yeast. Full length *RrMYB12* and *RrMYB111* was cloned into the pGBKT7 vector. The *RrMYB12*-BD and *RrMYB111*-BD construct and BD were transformed into Y2H yeast cells, respectively. The Y2H yeast cells transformed with *RrMYB12*-pGBDT7 and *RrMYB111*-pGBDT7 grown normally on SD/-Trp-His and appeared blue on SD/-Trp-His with X-α-Gal, while the negative control group showed an inhibition of colony growth (**Figure 2F**).

### Overexpression of *RrMYB12* and *RrMYB111* in tobacco promote flavonols and suppress anthocyanin accumulation accumulated in petals

**Figure 3:**
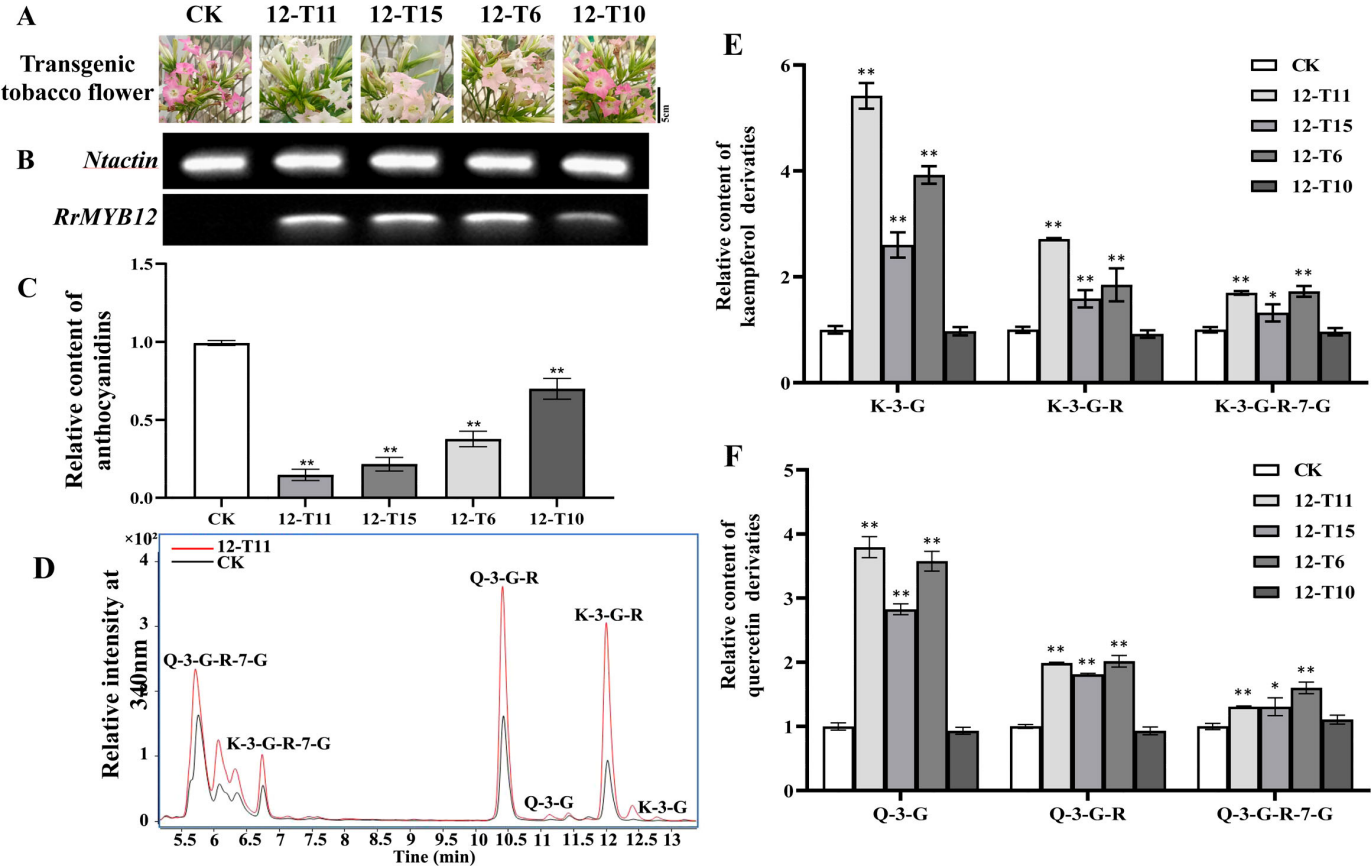
Analyses of *RrMYB12*-OE and CK tobacco flowers. **(A)** Phenotypes of CK and *RrMYB12-*OE tobacco flowers. **(B)** Semiquantitative RT-PCR analysis of the transcription levels of *RrMYB12* in *RrMYB12*-OE and CK tobacco flowers. **(C)** Relative content of anthocyanin extracts from CK and *RrMYB12-*OE tobacco flowers. **(D)** Total ion chromatogram of favonols in CK and T12-11 tobacco flowers. **(E)** Relative accumulation of kaempferol glycosides of CK and *RrMYB12-*OE tobacco flowers. **(F)** Relative accumulation of quercetin glycosides of CK and *RrMYB12-*OE tobacco flowers. Significant difference was indicated by the asterisk (*P < 0.05, **P < 0.01).

**Figure 4:**
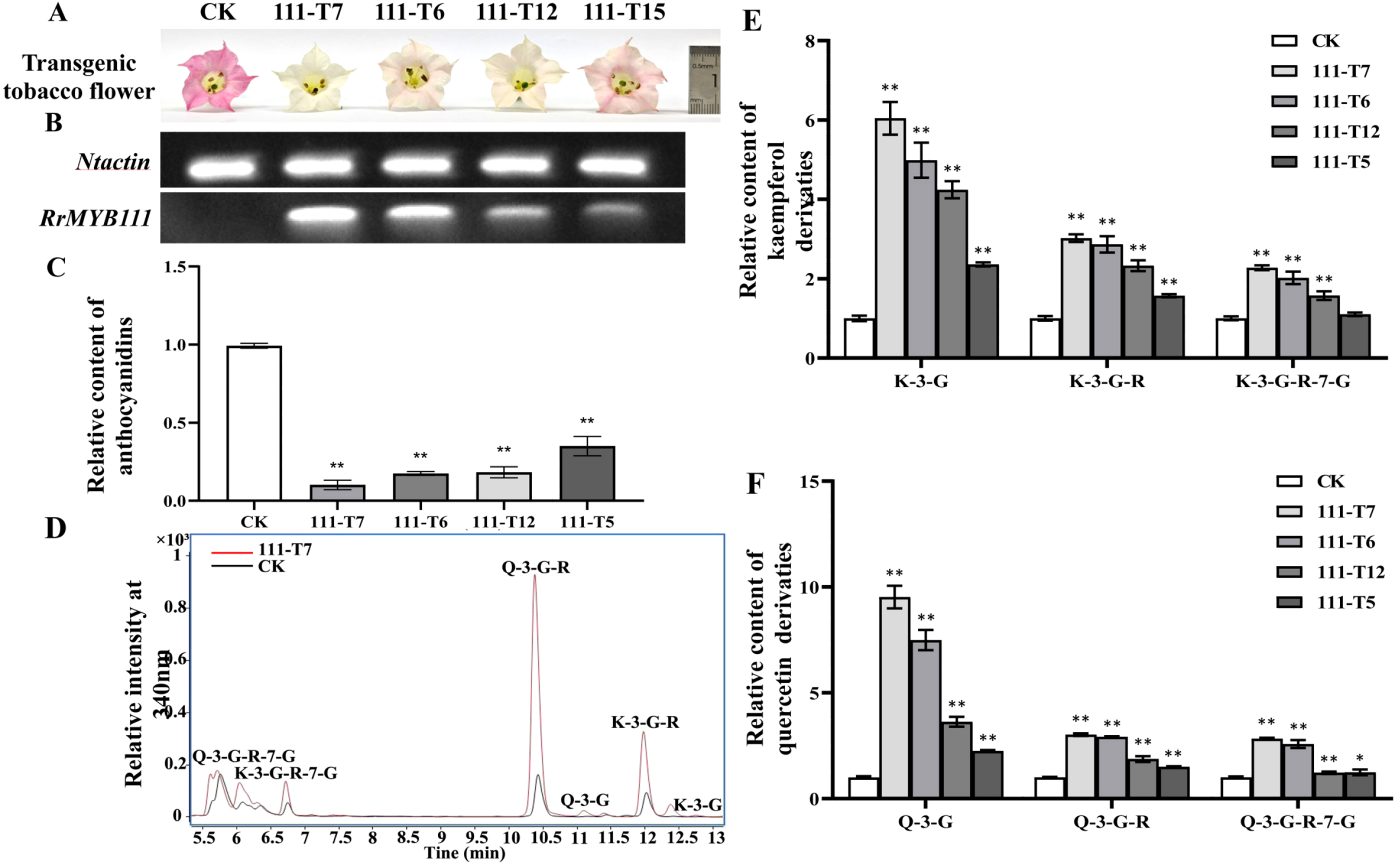
Analyses of *RrMYB111*-OE and CK tobacco flowers. **(A)** Phenotypes of CK and *RrMYB111-*OE tobacco flowers. **(B)** Semiquantitative RT-PCR analysis of the transcription levels of *RrMYB1111* in *RrMYB111*-OE and CK tobacco flowers. **(C)** Relative content of anthocyanin extracts from CK and *RrMYB111-*OE tobacco flowers. **(D)** Total ion chromatogram of favonols in CK and T111-7 tobacco flowers. **(E)** Relative accumulation of kaempferol glycosides of CK and *RrMYB111-*OE tobacco flowers. **(F)** Relative accumulation of quercetin glycosides of CK and *RrMYB111-*OE tobacco flowers. Significant difference was indicated by the asterisk (*P < 0.05, **P < 0.01).

The SG7 R2R3-MYB transcription factors were considered to regulate the accumulation of flavonols. To further investigate the function of *RrMYB12* and *RrMYB111* in flavonoid metabolism *in vivo*, stable heterologous overexpression of *RrMYB12* and *RrMYB111* was performed in tobacco plants. More than twenty transgenic tobacco lines were obtained for each construct. Transgenic tobacco plants containing the overexpression constructs of *RrMYB12* and *RrMYB111* exhibited normal growth. However, some of these transgenic plants produced white and light pink flowers compared to the control group plants (CK) (**Figures 3A**, **4A**). Semiquantitative analyses revealed significant levels of *RrMYB12* and *RrMYB111* mRNA corresponding to the observed changes in flower color (**Figures 3B**, **4B**).

Then, the flavonoid contents, including total anthocyanins and flavonol glycosides, were measured in transgenic tobacco flowers. Metabolic analysis conducted by spectrophotometry confirmed that the anthocyanin content extracted from *RrMYB12*-overexpressed (*RrMYB12*-OE) and *RrMYB111*-overexpressed (*RrMYB111*-OE) tobacco petals was significantly lower than that in CK (**Figures 3C**, **4C**). For instance, the anthocyanin contents in *RrMYB12*-OE line11 (T12-11) and *RrMYB111*-OE line17 (T111-7) accounted for only 14.69% and 10.13% of the anthocyanin contents in CK. The higher the expression of heterogeneously genes, the lower the accumulation of anthocyanins in transgenic tobacco flowers was observed as a phenomenon. Conversely, UPLC-MS analysis revealed that the levels of flavonols accumulated in *RrMYB12*-OE and *RrMYB111*-OE flowers were significantly higher compared to those found in CK flowers, with the exception of *RrMYB12*-OE line10 (T12-10), where there was minimal alteration observed in the content of flavonol glycosides. (**Figures 3D-F**, **4D-F**). For example, the measurements of K-3-G, K-3-G-R, and K-3-G-R-7-G in T12-11 increased to approximately 5.42, 2.71 and 1.70 times those of the control petals (**Figure 3E**). And the measurements of Q-3-G, Q-3-G-R, and Q-3-G-R-7-G in T111-7 increased to approximately 9.52, 3.02 and 2.83 times those of the control petals (**Figure 4F**).

**Figure 5:**
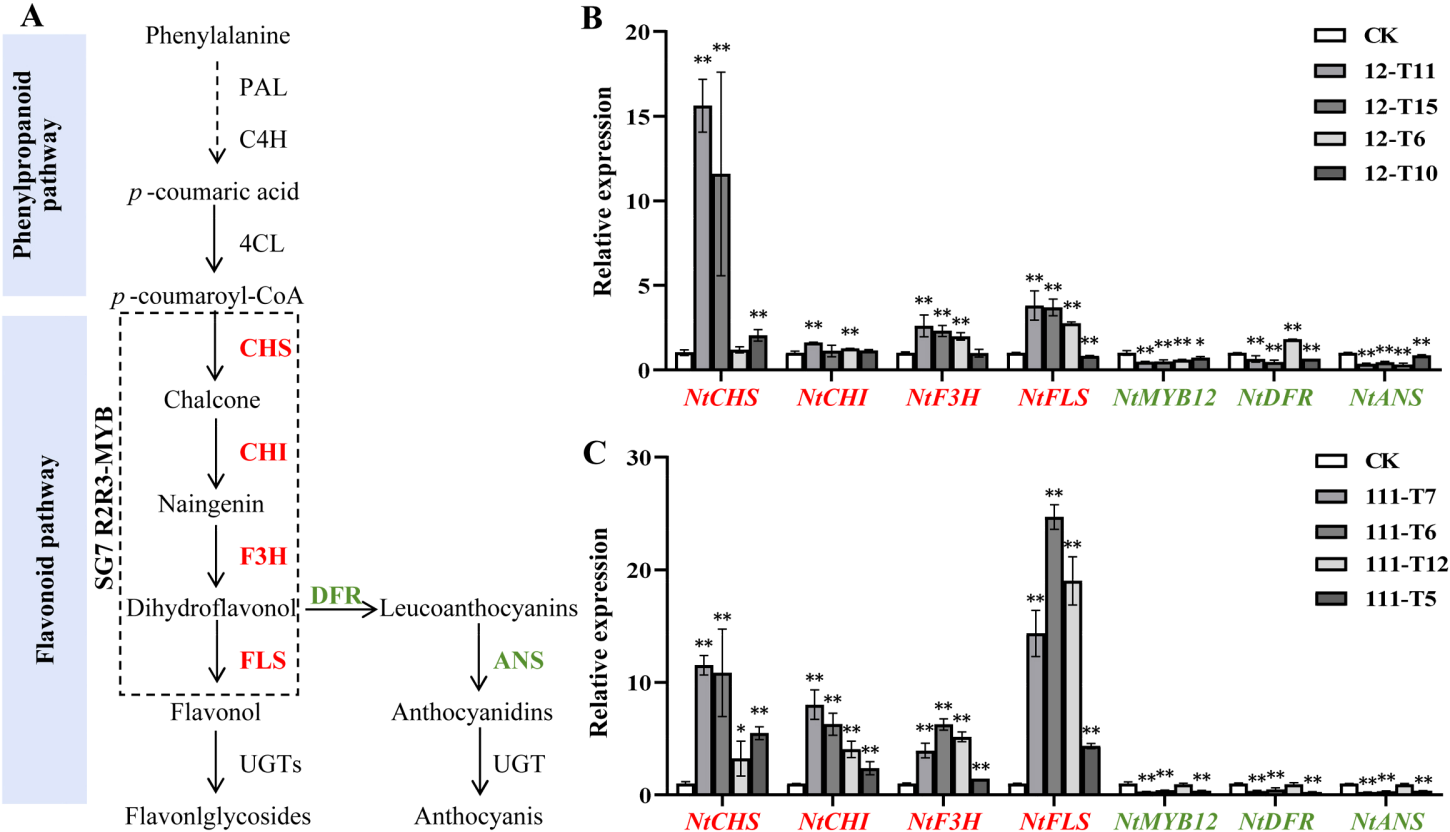
Analysis of the expression of flavonoid pathway genes in *RrMYB12*-OE and *RrMYB111*-OE tobacco flowers. **(A)** Schematic diagram of biosynthetic pathways of flavonols and anthocyanins in tobacco. **(B)** Relative expression of flavonoid biosynthetic pathway genes in *RrMYB12*-OE tobacco flowers by using the qRT-PCR analysis. **(C)** Relative expression of flavonoid biosynthetic pathway genes in *RrMYB111*-OE tobacco flowers by using the qRT-PCR analysis. Red text represents genes with increased expression and green text represents genes with decreased expression. Asterisks represent statistically significant differences (*P < 0.05, **P < 0.01).

The expression of *FLS* and *DFR* was considered to be strongly associated with the coloration of plants flowers (**Figure 5A**). The qRT-PCR method was conducted to examine the expression levels of endogenous genes involved in the flavonoid pathway in *RrMYB12*-OE and *RrMYB111*-OE flowers. The qRT-PCR results showed that *NtMYB12*, the tobacco homologous gene of *RrMYB12* and *RrMYB111*, was inhibited to varying degrees compared with CK. And the expression of *NtDFR* and *NtANS* genes associated with anthocyanin synthesis exhibited a significant reduction. The *NtCHS*, *NtCHI*, *NtF3H* and *NtFLS* genes, related to flavonol synthesis, were significantly upregulated in most *RrMYB12*-OE and *RrMYB111*-OE plants (**Figures 5A, B**).

These results suggest that the ectopic expression of *RrMYB12* and *RrMYB111* in tobacco redirects the metabolic flux from the anthocyanin pathway towards the flavonol pathway.

### RrMYB12 and RrMYB111 activate promoters of the flavonol pathway genes

**Figure 6:**
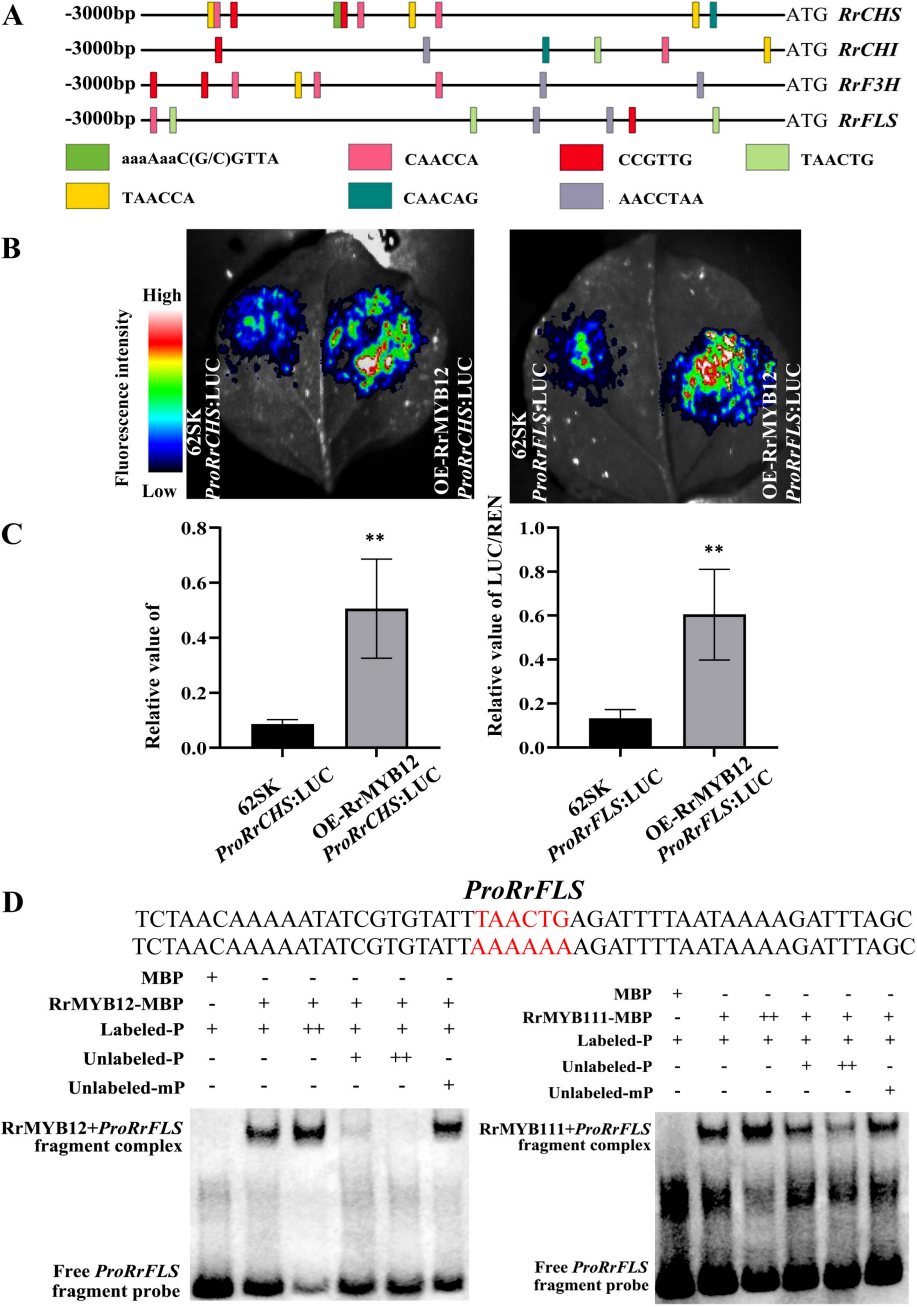
Analysis of regulation mechanism of flavonol biosynthesis pathway by RrMYB12 and RrMYB111. **(A)** The potential MYB binding sites in *RrCHS*, *RrCHI*, *RrF3H*, *RrFLS* promoters. **(B)** luminous images of RrMYB12 on activation of the *RrCHS* and *RrFLS* promoters in *N*. *benthamiana* leaves. Color scale indicates LUC signal intensity (red, strong; blue, weak). **(C)** The LUC/REN ratio of RrMYB12 on *RrCHS* and *RrFLS* promoters. Asterisks represent statistically significant differences (**P<0.01). **(D)** Detection of the binding ability of RrMYB12 and RrMYB111 to the *RrFLS* promoter by EMSA. The sequences of wild and mutant probes were presented, and the MYB-binding site was marked with red color. “-” represents absence, “+” represents presence, and “++” represents an excessive amount of the proteins and probes.

The expressions of *NtCHS*, *NtCHI*, *NtF3H* and *NtFLS* genes were more strongly increased than other genes of the flavonol pathway in *RrMYB12*-OE and *RrMYB111*-OE tobacco lines. We speculated that *RrCHS*, *RrCHI*, *RrF3H* and *RrFLS* may be potential targets of *RrMYB12* and RrMYB111 transcriptional activation. First of all, the promoter sequences of the *RrCHS*, *RrCHI*, *RrF3H* and *RrFLS* genes in roses were analysed by PlantCARE website. Meanwhile, the *NtCHS*, *NtCHI*, *NtF3H* and *NtFLS* promoters in tobacco were also analyzed (**Supplementary Figure 2**). According to the analysis, the sequences of *RrCHS*, *RrCHI*, *RrF3H* and *RrFLS* promoters all contain multiple MYB sites, such as CAACCA, TAACTG and TAACCA, etc., which are recognized by MYB transcription factors and serve as MYB binding sites (**Figure 6A**). This may suggests a direct regulatory role of RrMYBs on the structural genes involved in flavonol biosynthesis.The LUC and EMSA were carried out to determine the interaction of transcription factors and promoters.

Due to the duplication of MYB sites between the promoters in different structural genes, the promoter sequences of the *RrCHS* and *RrFLS* genes in roses were cloned. And the transcription activities of RrMYB12 and RrMYB111 against the *RrCHS* and *RrFLS* promoters were detected using a dual-luciferase reporter assay by transiently transformed leaves of *N*. *benthamiana* respectively. The RrMYB12 with *proRrCHS* or *proRrFLS* exhibited signifcantly higher LUC activity compared with *proRrCHS* or *proRrFLS* alone when transiently expressed in tobacco leaf epidermal cells (**Figure 6B**). The activities were indicated by the ratio of LUC/REN. The luminescence intensities of *RrCHS* and *RrFLS* promoters were significantly increased by RrMYB12 in comparison to the corresponding controls that lacked RrMYB12 (**Figure 6C**). For example, The promoters of *RrFLS* were strongly activated up to 4.5 fold by RrMYB12. But these promoters were not activated by RrMYB111 in the transient reporter assay (**Supplementary Figure 3**).

To investigate whether the RrMYB12 and RrMYB111 proteins can directly bind to the promoter region of the flavonol metabolic-related gene *in vitro*, electrophoretic mobility shift assays were performed. As shown in **Figure 6D**, both the RrMYB12 and RrMYB111 protein can directly bind to the fragment of the *RrFLS* promoter. And the unlabeled probe competed with the biotin-labeled probe for binding to *RrMYB12*-pMAL and *RrMYB111*-pMAL. Moreover, excessive cold probes could obviously reduce the band shift, while mutant probes failed to compete with the binding of *proRrFLS*, indicating that the binding of RrMYBs to the *RrFLS* promoter region was specific. These results indicate that RrMYB12 and RrMYb111 specifically and directly bind to the TAACTG site in the *RrFLS* promoters.

## Discussion

The functional similarity of R2R3-MYB TFs can be predicted by their structural similarity. The amino acid sequence of RrMYB12 and RrMYB111 revealed the presence of the highly conserved R2R3 domain at the N-terminus, which is characteristic of MYB transcription factors (**Figure 1B**). The SG7 motif, which is characteristic of flavonol biosynthesis regulators before, was identified at the C-terminus of both RrMYB12 and RrMYB111 (**Figure 1B**) (Czemmel et al., 2009; Stracke et al., 2007). And the SG7-2 motif in the C-terminal region was only identified at RrMYB12 not identified at RrMYB111 which is similar to the amino acid sequence characteristics of AtMYB12 and AtMYB111 (**Figure 1B**) (Stracke et al., 2007). Additionally, the R2 and R3 domains of RrMYB12 and RrMYB111 does not contain the conserved bHLH interaction motif identified previously, suggesting that RrMYB12 and RrMYB111 is bHLH cofactor independent (Zhang et al., 2023; Zhao et al., 2023; Zimmermann et al., 2004). This cofactor independency is generally restricted to the MYB factors of the flavonol clade (Huang et al., 2016). Phylogenetic analysis indicated that RrMYB12 and RrMYB111 were clustered within subgroup 7, with other known flavonol regulators such as AtMYB11, AtMYB12, and AtMYB111 from *A. thaliana*, LjaMYB12 from *L. japonica* and VvMYBF1 from *V. vinifera* (**Figure 1C**). All of these indicated that RrMYB12 and RrMYB111 should be a potential R2R3-MYB protein that has the function of regulating flavonol biosynthesis.

As known, the expression of R2R3-MYB genes in the flavonol pathway, such as *AtMYB12* and *VvMYBF1*, exhibits a strong correlation with flavonol synthesis and accumulation (Czemmel et al., 2009; Stracke et al., 2007). Therefore, analyzing the gene expression of *RrMYB12* and *RrMYB111* would be helpful for understanding the pattern of flavonol accumulation in *R. rugosa*. The qRT-PCR analysis revealed that *RrMYB12* exhibited high expression in leaf and low expression in root and flower, while the expression of *RrMYB111* in bud and leaf was almost the same (**Figures 2A-C**). And in different parts of rose flower, *RrMYB12* was highly expressed in the calyx, while *RrMYB111* was highly expressed in the pistil (**Figures 2D, E**). These differential expression of genes in rose tissues is similar to *A. thaliana* or other plants that contain multiple SG7 MYB genes that the homologous genes showed spatial and temporal differences (Stracke et al., 2007). Based on these datas, it could be inferred that the flavonol contents in the stem, root, and flower of rose plant may be relatively low and the accumulation of flavonols in various parts of rose flower would be likely to be very complex. These datas and inferences can provide a basis for future molecular techniques to modify the accumulation of rose flavonoids in different tissues.

The regulatory role of SG7 R2R3-MYB in flavonol synthesis is highly conserved across diverse plant species. In addition to demonstrating the function of SG7 R2R3-MYB in regulating flavonol synthesis by multiple mutants of *atmyb12*, *atmyb111* and *atmyb11*, similar results were also obtained when overexpressing *SG7 MYB* genes in the heterologous system (Czemmel et al., 2009; Stracke et al., 2007). For example, The expression of *AtMYB12* in tobacco resulted in a several-fold increase in flavonol accumulation in tobacco, as well as in tomato enhanced flavonol accumulation in tomato leaves and fruits (Ashutosh et al., 2015; Prashant et al., 2010). And the ectopic expression of *PqMYBF1* in transgenic tobacco also caused an observable elevation in flavonol levels (Zhang et al., 2023). In this study, the introduction of *RrMYB12* and *RrMYB111* overexpression constructs into tobacco plants resulted in a significant enhancement of flavonol accumulation in the majority of *RrMYB12*-OE and *RrMYB111*-OE tobacco lines (**Figures 3**, **4**). These findings suggest that both *RrMYB12* and *RrMYB111* can play a role in the regulation of flavonol biosynthesis in tobacco flowers.

In most plant species, anthocyanins and flavonols are synthesized within the same cell and typically accumulate in the same subcellular location. Thus, potential competition may exist between anthocyanins and flavonols. In fact, the overexpression of *GhMYB1a* resulted in an excessive accumulation of kaempferol, while significantly reducing petal pigmentation during flower development (Zhong et al., 2020). Therefore, we determined the anthocyanin content in both the transgenic flowers and CK flowers. Compared to the flowers of CK, both *RrMYB12*-OE and *RrMYB111*-OE transgenic tobacco exhibited a significant reduction in anthocyanin content(**Figures 3C**, **4C**).

The inverse correlation observed between anthocyanin and flavonol levels in the SG7 R2R3-MYB genes-overexpressing plant lines likely reflects a competitive relationship between these two branches of flavonoid metabolites. Previous studies have demonstrated that ectopic expressions of these MYB regulators in tobacco or tomato can modulate the genes involved in the flavonol pathway, resulting in enhanced flavonol biosynthesis and consequently inhibiting the accumulation of anthocyanins. For example, flavonol pathway genes, including *NtCHS*, *NtCHI*, *NtF3H* and *NtFLS* can be generally upregulated and the anthocyanin biosynthetic pathway genes, *NtDFR* and *NtANS* were not significantly modulated or downregulated in transgenic tobacco by overexpressing *PqMYBF1* or *EsMYBF1* (Huang et al., 2016; Zhang et al., 2023). As reported, AtMYB12 mainly targets *AtCHS* and *AtFLS* genes, AtMYB11 and AtMYB111 could increase the expression of the *AtCHS* gene in *A. thaliana* to 36 and 399 times, respectively (Stracke et al., 2007). Yang et al. found that overexpression of *CmMYB3* form *Chrysanthemum morifolium* could efectively promote the expression of these four genes related to favonol biosynthesis in *A. thaliana*, especially the expression level of *AtCHS* and *AtFLS* genes increased by more than 10 times (Yang et al., 2023). Similarly, our research also showed that in *RrMYB12*-OE and *RrMYB111*-OE tobacco lines, the expression of *NtCHI*, *NtCHS*, *NtF3H* and *NtFLS* was upregulated, especially *NtCHI* and *NtFLS* more than 15 times, and the expression levels of *NtMYB12*, *NtDFR* and *NtANS* were significantly decreased (**Figure 5**). The results suggest that RrMYB12 and RrMYB111 exert direct influence on flavonol accumulation primarily by regulating the expression of key genes involved in the flavonoid pathway. Additionally, they cause competitive inhibition of *NtMYB12* and *NtDFR* and *NtANS*. Overall, the present findings collectively provide compelling evidence supporting the functional regulation of flavonol synthesis by RrMYB12 and RrMYB111.

The mechanism of R2R3-MYB transcription factors in the regulation of flavonol and anthocyanin biosynthesis has been extensively studied. Previous studies have shown that SG7 MYB promoted favonol biosynthesis by regulating more than one gene expression in the secondary metabolic pathway (Yang et al., 2023). PqMYBF1 have been reported to activate the flavonol pathway genes *PqCHS*, *PqF3H*, and *PqFLS* independently of a bHLH cofactor (Zhang et al., 2023). However, the SG7 R2R3-MYB transcription factor, CmMYB9a, activates foral coloration in chrysanthemum by positively regulating *CmCHS*, *CmDFR* and *CmFNS*, but inhibiting the expression of *CmFLS* (Wang et al., 2021). So, even in the same family, the functions of diferent members sometimes may have signifcant diferences. And our study found that the RrMYB12 and RrMYB111 proteins activate the flavonol pathway genes independently. As shown in **Figure 2F**, yeast cells containing the *RrMYB12* and *RrMYB111* could grow well and turn blue on SD/-Trp-His medium with X-α-Gal, indicating that that RrMYB12 and RrMYB111 were the transcriptional activator. It was demonstrated that RrMYB12 can directly and specifically activate the flavonol pathway genes in the dual-luciferase reporter assay. Both the *RrCHS* and *RrFLS* promoters were significantly activated by RrMYB12 (**Figure 6B**), suggesting that RrMYB12 may be able to control the entire pathway leading to flavonol synthesis. In addition, EMSA results also showed the direct and specific binding of RrMYB12 to the MYB site of *RrFLS* promoter (**Figure 6C**). These results suggest that RrMYB12 specifically activates the flavonol pathway genes. As for RrMYB111, why were the *RrCHS* and *RrFLS* promoters not activated by RrMYB111 in the LUC experiment? Some researches could help us speculate that RrMYB111 may be a partially atypical SG7 R2R3-MYB protein and the SG7-2 motif was speculated to might affect the regulatory function on flavonol biosynthesis of SG7 MYB transcription factors (Wang et al., 2017; Yang et al., 2023; Zhou et al., 2021). Whether the absence of SG7-2 motif of the RrMYB111 protein is related to the inability of *RrCHS* and *RrFLS* promoters to be activated need further study.

Our findings not only provide further understanding of the regulation of the flavonol biosynthetic pathway in roses but also highlight a potential regulator that could be utilized for genetic manipulation to regulate pigmentation accumulation.

## Data Availability

The original contributions presented in the study are included in the article/**Supplementary Material**. Further inquiries can be directed to the corresponding author.

## Supplementary material

Supplementary Table 1 Primer sequences used for *RrMYB12* and *RrMYB111* in this study.

Supplementary Table 2 Primer sequences used for promoters in this study.

Supplementary Table 3 Sequences used for EMSA probe synthesis in this study.

Supplementary Table 4 Primer sequences used for qRT-PCR analysis in this study.

Supplementary Figure 1 Schematic diagram of conservative analysis of promoter insertion sites.

Supplementary Figure 2 The potential MYB binding sites in *NtCHS*, *NtCHI*, *NtF3H*, *NtFLS* promoters by PlantCARE.

Supplementary Figure 3 Transcriptional activity assay of RrMYB111 on promoters of rose flavonoid-related genes. **(A)** The LUC/REN ratio of RrMYB111 on *RrCHS* promoter. **(B)** The LUC/REN ratio of RrMYB111 on *RrFLS* promoter.
