# Supplementary figures and images for: Rosa rugosa R2R3-MYB transcription factors RrMYB12 and RrMYB111 regulate the accumulation of flavonols and anthocyanins
Source: Front Plant Sci. 2024 Dec 17;15:1477278. doi: 10.3389/fpls.2024.1477278 (PMC11685124; doi:10.3389/fpls.2024.1477278)

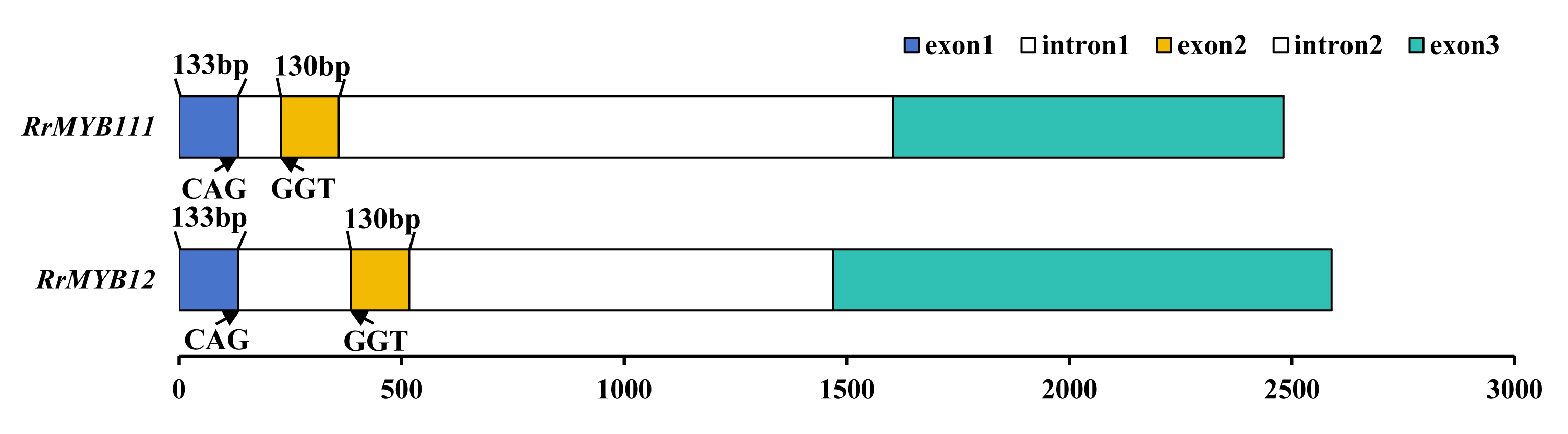

Supplement: Supplementary Figure 1 — Schematic diagram of conservative analysis of promoter insertion sites. [file Image1.jpeg]

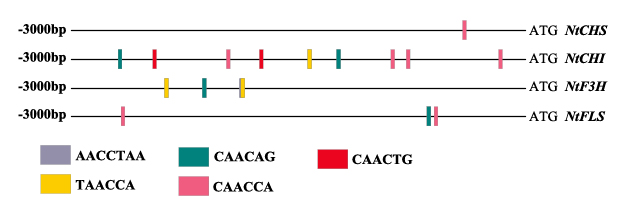

Supplement: Supplementary Figure 2 — The potential MYB binding sites in NtCHS, NtCHI, NtF3H, NtFLS promoters by PlantCARE. [file Image2.jpeg]

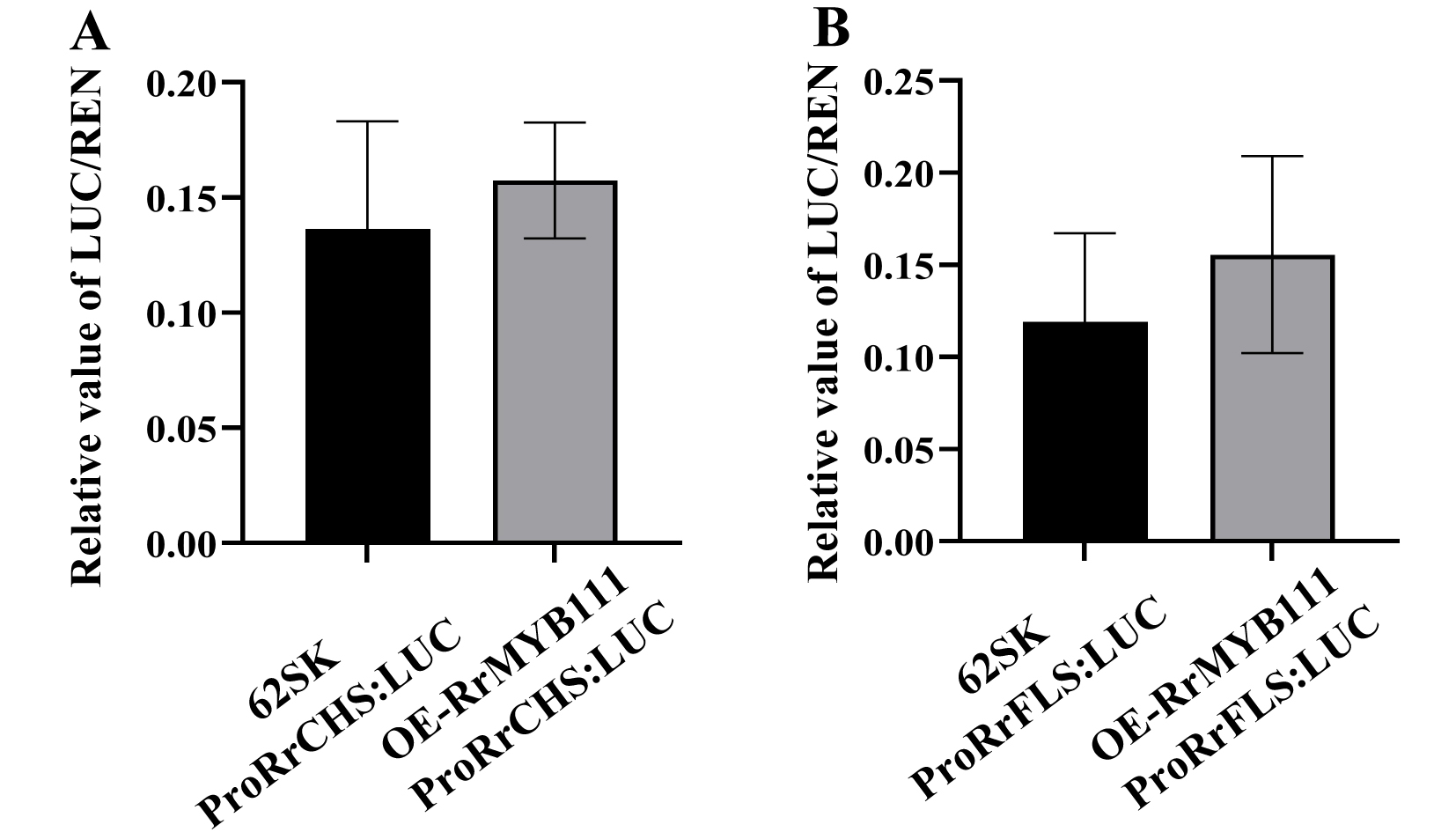

Supplement: Supplementary Figure 3 — Transcriptional activity assay of RrMYB111 on promoters of rose flavonoid-related genes. (A) The LUC/REN ratio of RrMYB111 on RrCHS promoter. (B) The LUC/REN ratio of RrMYB111 on RrFLS promoter. [file Image3.jpeg]
